# Supplementary material for: Extended Energy-Expenditure Model in Soccer: Evaluating Player Performance in the Context of the Game
Source: Sensors (Basel). 2022 Dec 14;22(24):9842. doi: 10.3390/s22249842 (PMC9781885; doi:10.3390/s22249842)
Supplement: Supplementary file 1 [file sensors-22-09842-s001.zip › sensors-2008217-supplement.pdf]

Supplementary Materials: Extended Energy-Expenditure Model in Soccer: Evaluating Player Performance in the Context of the Game

Arian Skoki [1], Alessio Rossi [2,3,*], Paolo Cintia [2], Luca Pappalardo [2,3] and Ivan Štajduhar [1,4]

1. MFit Methodology

Once we classify every minute of players' game effort according to energy expenditure (clusters), we can estimate the ability to sustain repeated high-intensity efforts during the game. The intuition behind MFit is: the more a player is able to keep repeating high-intensity effort during a game, the higher is his fitness. Information about the ability of a player to sustain a high working load and the required time needed to recover can be inferred by looking at consecutive high-intensity periods. To do that, clusters for a particular player and game were converted into a dataset containing the name of the cluster, duration of the cluster, start time, and end time. This enabled counting of transition states between the low-, middle-, and high-intensity periods. The importance in this is the ability to show how likely a player is to keep performing at the highest intensity, given that his previous state (minute) was also high-intensity. What we have just explained is a base for a transition matrix in the Markov chain. From the matrix, it is possible to obtain information about the transitions' probability between states. In particular, players' ability to perform multiple consecutive high-intensity periods (MFit value) will give information about the fitness status of both players and the team, and enable comparisons between them. Actually, for this aim, a transition probability matrix was built according to the number of occurrences (minutes) of a specific transition divided by the number of minutes in the game. To eliminate the influence of only one game, which could be lower or higher intensity than usual, several games should be taken into consideration. Otherwise, there will be a lot of deviation in the data that was caused by the different game demands. The other problem lies in players' playing minutes. In a single game, one can enter as a substitute, while in another he is in the starting eleven. That influences his ability to perform in the high-intensity zone. The assumption is that the substitutes can produce more high-intensity transitions per-minute-played than the eleven starting players.

To cope with the stated problems, an MFit analysis was done using 5 games that a player had played. For a general estimate of the players' or team's state, which will smooth the between-game differences, a rolling mean across the 5-game period was introduced. This was done by using each game's probability matrix, more specifically—the probability of remaining in the high-intensity zone. Thus, probabilities for each player were realistic, but they needed to be put into context.

An action in soccer can vary from a very short one (measured in seconds) to a combined team pressure that lasts for minutes. The general view is: if the team continues to press, it will create chances and eventually score a goal. This is the reason why we opted for 5- and 10-minute periods to serve as a test about players' ability to sustain high-intensity efforts. If a player can often sustain repeated high-intensity periods, his MFit index will be higher. The MFit-5 index is calculated by potentiating high-to-high transition probability with 5. This measure is suitable for most of the teams because they would need some rest time after an intense 5-min period. However, if the team plays high-possession style, then MFit-10 index might be more appropriate for assessment. It is measured in a similar manner, but the high-to-high transition probability is potentiated with 10.

Both MFit-5 and MFit-10 were calculated for all the players in the study. This was done by using rolling probabilities across the 5 games with a sliding window of 1. Additionally, all the players' probabilities were normalized by dividing them by the team probabilities, i.e., the matrix of all players in the dataset. The resulting values represented a player's fitness using MFit.

2. MFit Analysis: Tracking Players' Fitness Status

By using the proposed method of clustering, it is possible to better describe individual players' intensities and see the areas in which they may be lacking, such as stamina. Additionally, the data from all the players can be accumulated to acquire summed numbers of high-, middle-, and low-intensity minutes within the game. An energy-based approach, without the need to set thresholds, enables tracking both player and team load in each game. Actually, it provides an opportunity to track individual players' and teams' physical progress and fitness status.

Players' physical state can be tested by looking at the maximum number of high-intensity repetitions for particular players. However, looking only at the maximum values could lead to many spikes in the data, which is not a realistic representation of the player's physical condition. Further, the duration for which the player has made the maximum-intensity consecutive repetitions highly affects the result. A period that is longer induces a lack of sensitivity, and a too-short period would be highly context-dependent. For the named reasons, the number of games and minutes played need to be taken into consideration to compare games by the same player or different players. As a matter of fact, a player that always played as a substitute may have never reached a high-intensity count because of not having enough in-game time. An example of the maximal number of repetition analyses for a player throughout the soccer season can be seen in Figure S1.

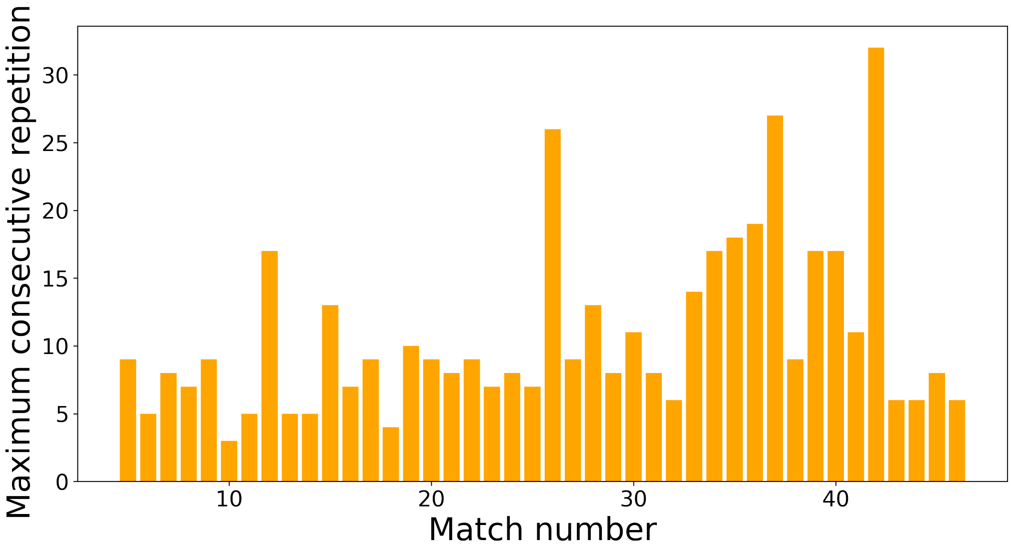

Figure S1. Maximum high-intensity repetitions per game for a particular player.

Additionally, the transition repetition matrix was created to test the players' ability to sustain high-intensity in the periods of 5 minutes (MFit-5) and 10 minutes (MFit-10), with an interest in evaluating their fitness. These metrics indicate a probability that a player will perform 5 (MFit-5) or 10 (MFit-10) consecutive high-intensity periods with respect to other players on the team. An example of a probability matrix for a player and a game (used in Figure 7 of the main paper) is shown in Figure S2. In this plot, it is possible to detect that this player, when the clustering classification was low-intensity, had a similar probability to remain in the same group (30.77%) and a slightly higher probability to traverse to the middle- and high-intensity groups (30.77% and 38.46%, respectively). Alternatively, when the player was allocated to either the middle- or the high-intensity group, the most probable outcome was to remain in the same group. Additionally, the results of MFit-5 and MFit-10 minutes high-intensity repetition probability for CB, MF, and FW can be seen in Figures S3 and S4, respectively.

The final part of the repeatability analysis shows its power by comparing it to the most commonly used GM-GAME metrics: total distance (m) and HSR distance (m) (see

Figure S5). In this analysis, HSR and sprint distance were summed up together to provide a more realistic number for the peak intensity. Next, these GM-GAME values were calculated in the same way as the repeatability analysis—by applying a rolling mean across the 5 games—and the result can be seen in Figures S5a,b. Total and HSR running distance provide information about the game volume but are less informative concerning other aspects, such as energy expenditure, number of accelerations and decelerations, time spent in recovery, and so on. Therefore, it is impossible to look at the whole picture by using only these values. The combination of additional GM-GAME parameters surely provides more information and can be used for analyzing the fitness of the players. However, this analysis would be complicated for the practitioner as it would require tracking multiple metrics at the same time. Unfortunately, there is no single metric that can be compared with the MFit value. Only the analytical approach with trends is used, similar to the STATSports article regarding the macrocycle overview [1]. The methods proposed in this study provide a single value—the MFit index—that combines many parameters and enables one to track the players' fitness more easily. The main advantage of MFit index is the ability to express multiple features in a single value without the need for slow parameter selection and evaluation. The whole processing approach provides a platform on which additional insights can be built.

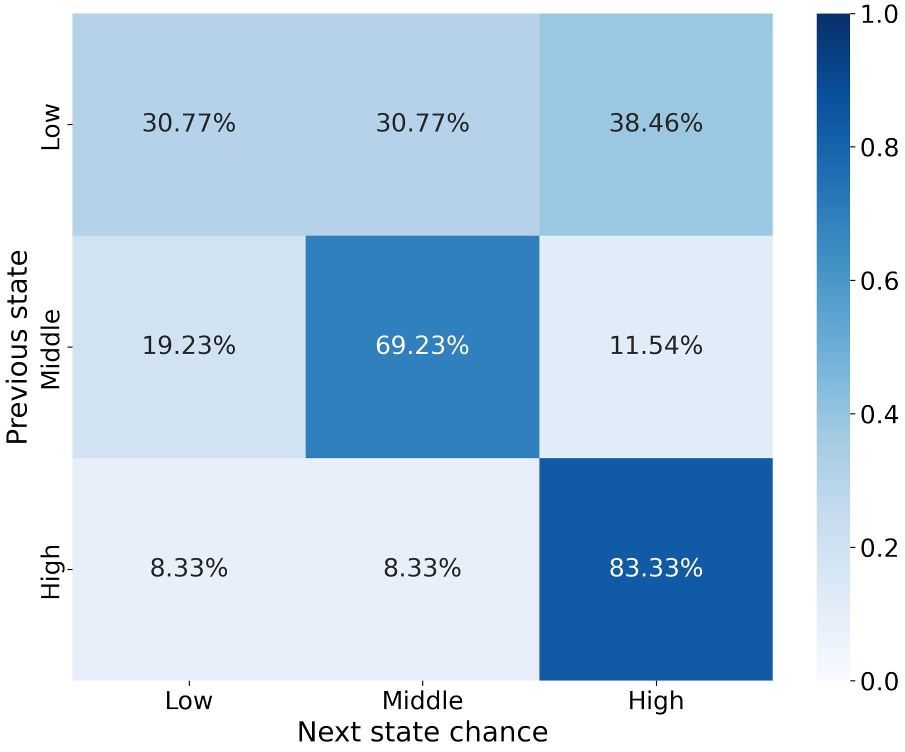

Figure S2. Transition probability matrix used in MFit analysis for the example shown in Figure 7 of the main paper. The player has a very high possibility (83.33%) of remaining in the high-intensity zone given that he is already performing in the high-intensity zone.

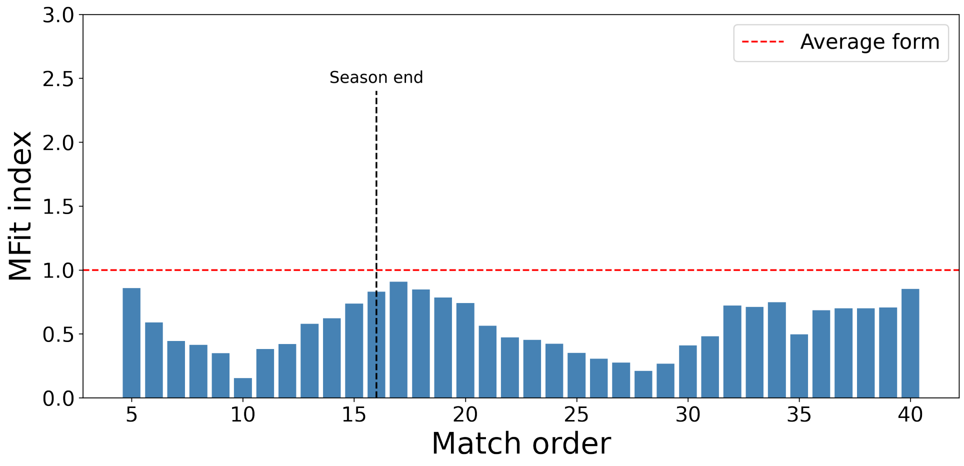

(**a**) MFit-5 for a single player in a CB playing position.

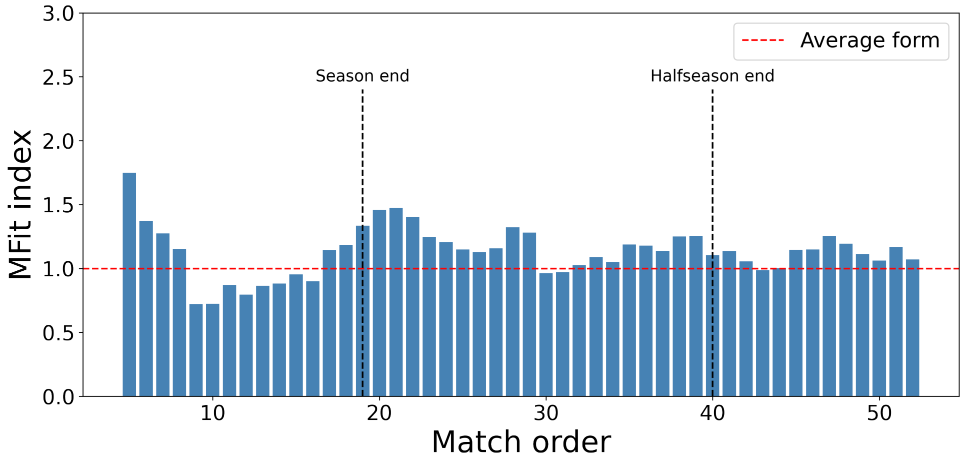

(**b**) MFit-5 for a single player in an MF playing position.

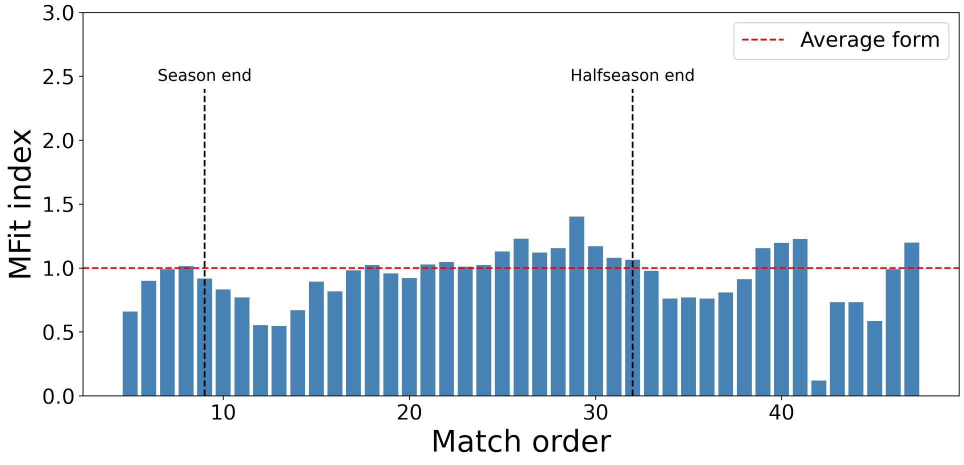

(**c**) MFit-5 for a single player in an FW playing position.

Figure S3. MFit-5 through the season, made with a 5-min high-intensity repetition probability. An example is provided for each role, based on a single player. The red dotted line refers to the probability repetition average of all the players in the dataset.

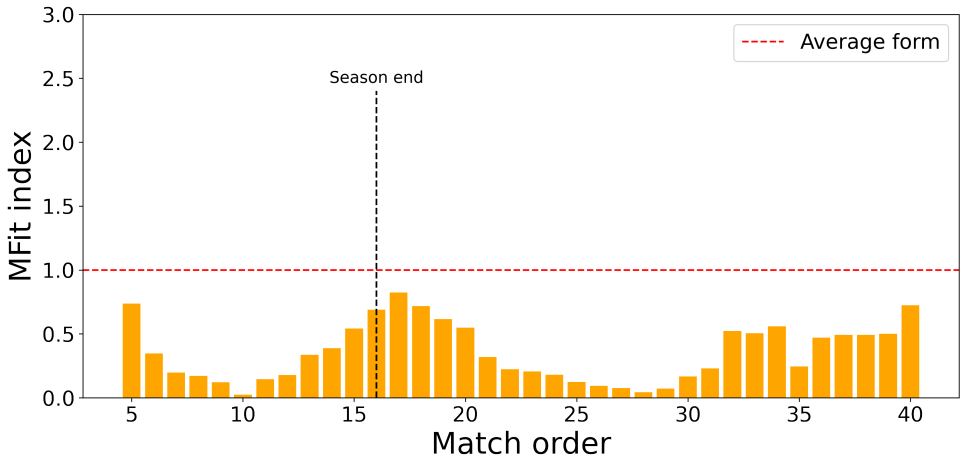

(**a**) MFit-10 for a single player in a CB playing position.

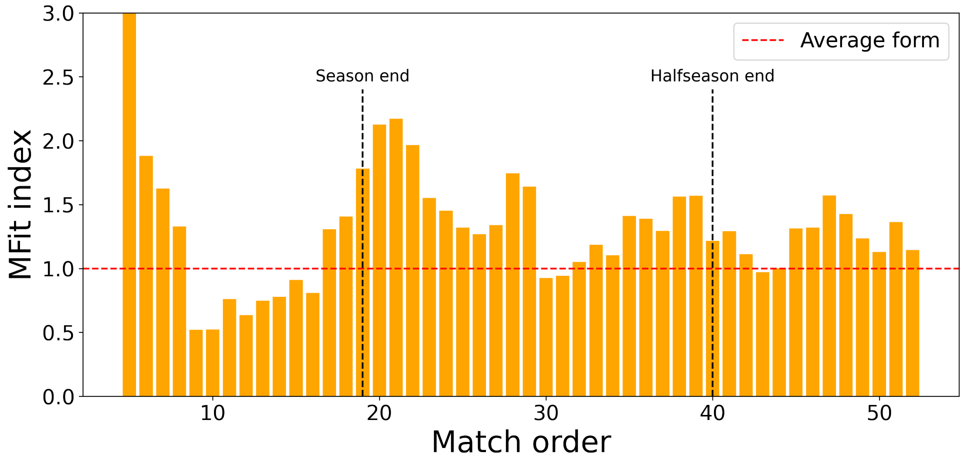

(**b**) MFit-10 for a single player in an MF playing position.

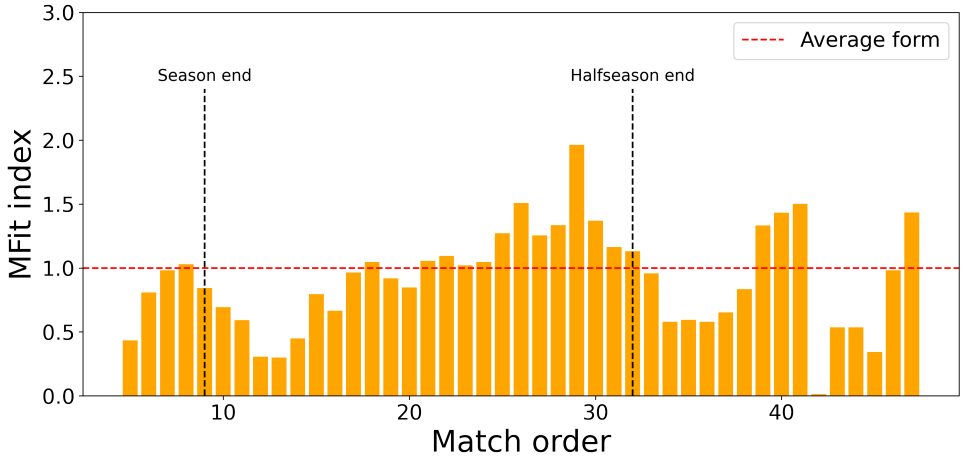

(**c**) MFit-10 for a single player in an FW playing position.

Figure S4. MFit-10 through the season, made with a 10-min high-intensity repetition probability. An example is provided for each role, based on a single player. The red dotted line refers to the probability repetition average of all the players in the dataset.

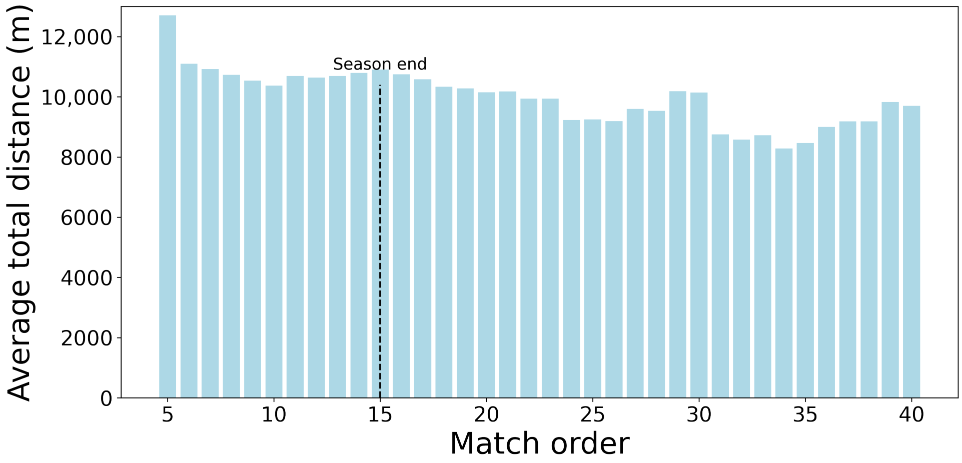

(**a**) Total distance average (m) across games for a single player in a CB playing position.

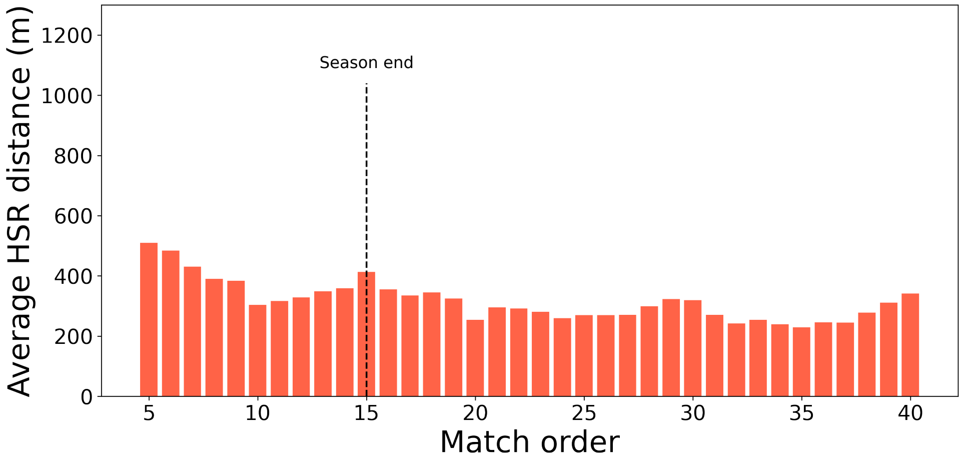

(**b**) HSR distance average (m) across games for a single player in a CB playing position.

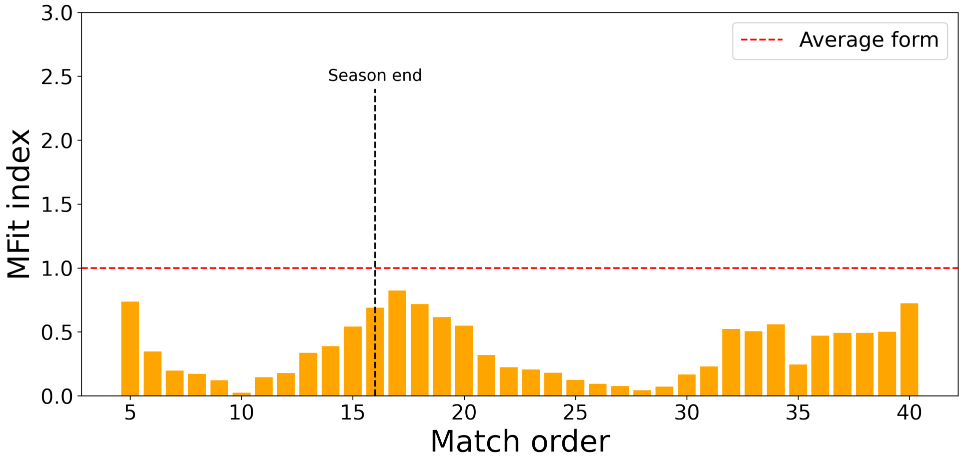

(**c**) MFit-10 for a single player in a CB playing position.

Figure S5. Analytical comparison between widely used GM-GAME and a MFit-10 for a player in CB position. GM-GAME features show very low variability and, therefore, an inability to express player physical effort differences on a game-to-game basis.

References

1. Cian, C. Macrocycle Overview—Does Player Tracking aid Periodized Peak Performance? Available online: https://pro.statsports.com/macrocycle-overview-does-player-tracking-aid-periodized-peak-performance/ (accessed on 1 December 2022)
